# Diagnostic performance of pupil perimetry in detecting hemianopia under standard and virtual reality viewing conditions

**DOI:** 10.1007/s00417-024-06641-4

**Published:** 2024-09-18

**Authors:** Brendan Portengen, Saskia Imhof, Marnix Naber, Giorgio Porro

**Affiliations:** 1 Ophthalmology Department, University Medical Center Utrecht, Room E 03.136, PO Box 85500, 3508 GA Utrecht, The Netherlands; 2 Experimental Psychology, Helmholtz Institute, Utrecht University, Utrecht, The Netherlands

**Keywords:** Perimetry, Pupillometry, Visual field, Scotoma, Virtual reality

## Abstract

**Purpose:**

To determine the diagnostic performance and reliability of two pupil perimetry (PP) methods in homonymous hemianopia.

**Methods:**

This cross-sectional monocenter cohort study performed gaze-contingent flicker PP (gcFPP) and a virtual reality version of gcFPP (VRgcFPP) twice on separate occasions in all patients suffering from homonymous hemianopia due to neurological impairment. The main outcomes were (1) test accuracy and (2) test–retest reliability: (1) was measured through area under the receiver operating characteristics curve (AUC) calculation of (VR)gcFPP results with comparators being SAP and healthy controls, respectively; (2) was evaluated by comparing tests 1 and 2 of both methods within patients.

**Results:**

Both gcFPP and VRgcFPP were performed in 15 patients (12 males, M_Age_ = 57, SD_Age_ = 15) and 17 controls (6 males, M_Age_ = 53, SD_Age_ = 12). Mean test accuracy was good in separating damaged from intact visual field regions (gcFPP: M_auc_ = 0.83, SD_auc_ = 0.09; VRgcFPP: M_auc_ = 0.69, SD_auc_ = 0.13) and in separating patients from controls (gcFPP: M_auc_ = 0.92, SD_auc_ = 0.13; VRgcFPP: M_auc_ = 0.96, SD_auc_ = 0.15). A high test–retest reliability was found for the proportion intact versus damaged visual field (gcFPP: *r* = 0.95, *P* < .001, VRgcFPP: *r* = 1.00, *P* < .001).

**Conclusions:**

Overall, these results can be summarized as follows: (1) the comparison of pupil response amplitudes between intact versus damaged regions per patient indicate that gcFPP allows for cleaner imaging of intact versus damaged visual field regions than VRgcFPP, (2) the comparisons of average differences in intact versus damaged amplitudes between patients and controls demonstrate high diagnostic performance of both gcFPP and VRgcFPP, and (3) the test–retest reliabilities confirm that both gcFPP and VRgcFPP reliably and consistently measure defects in homonymous hemianopia.

**Key  messages:**

***What is known***

- Standard automated perimetry is the current gold standard for visual field examination, but not always suited for the evaluation of the VF in neurologically impaired patients.
- Pupil perimetry consists of the measurement of pupillary responses to light stimuli as a measure of visual sensitivity.

***What is new***

- This study reports the highest diagnostic accuracy of pupil perimetry so far in patients with homonymous hemianopia.
- Gaze-contingent flicker pupil perimetry reliably and consistently measures defects in homonymous hemianopia under standard and virtual reality viewing conditions.

**Supplementary Information:**

The online version contains supplementary material available at 10.1007/s00417-024-06641-4.

## Introduction

Standard automated perimetry (SAP) is the current gold standard for visual field (VF) examination, but not always suited for the evaluation of the VF in neurologically impaired patients [1]. Neurologic VF defects (VFD) (e.g., homonymous hemianopia) result in bilateral hemifield loss on the side contralateral to the underlying lesion, typically respecting the vertical midline. Such VF loss is relatively easy to simulate [2]. Moreover, SAP methods suffer from high test–retest variability [3, 4]. The poor reproducibility might be caused by retinotopic displacement of small stimuli due to the fixational jitter or microsaccades, task learning effects, and fatigue effects [5–7], which are especially problematic in patients with neurological impairment. There are few alternative solutions that address the above-described problems.

One alternative is pupil perimetry (PP; see [8]), which consists of the measurement of pupillary responses to light stimuli as a measure of not only retinal sensitivity, but also visual sensitivity, which depends on the degree an observer pays visual attention to and is aware of light stimuli [9–11]. Simply put, pupil responses are strong when a stimulus is shown in the intact VF and weak or absent when the damaged VF is stimulated. As early as 1975, the PP method has been hypothesized to have merit in the assessment of individuals with neurological impairment due to its simple, noninvasive and objective nature [12]. Over the years, multiple iterations of PP have tried to improve diagnostic performance in this patient population [13–20]. To the best of our knowledge, gaze-contingent flicker PP (gcFPP) reports the highest diagnostic performance in neurologically impaired patients [20], and since this initial publication, improvements of this method have been tested on healthy controls [21–23]. These improvements included (1) adding color contrast between stimulus and background to evoke stronger pupil responses, (2) scaling stimuli as a function of eccentricity to take into account the cortical magnification factor, and (3) enhancements to pupil response analyses such as better on-line blink detection and trial repetitions rather than trial rejections. However, whether these improvements have led to improvements in diagnostic performance remains to be validated in patients.

Another development in the gcFPP method is the novel implementation of a head-mounted device with virtual reality (VR) technology, dubbed VRgcFPP [23]. VR applications in the ophthalmic practice are relatively new, but promising [24–31]. Particularly, VR seems to be preferred over screen-based approaches by patients [32]. VRgcFPP has shown merit in (healthy) young children due to its free range of movement, engaging visual task, and reliable pupil measurements with a built-in eye tracker [23]. Its ability to assess the visual field, however, has not yet been evaluated in patients with homonymous hemianopia. Also, while the core features of (VR)gcFPP (i.e. short test duration, no subjective response is needed and the larger stimuli are presented in a gaze-contingent manner) could produce a better reproducibility than SAP, its test–retest reliability of has so far not been studied.

The current study aims to fill the above-mentioned gaps by exploring the diagnostic performance of an improved version of pupil perimetry (i.e., gcFPP) and a novel virtual reality-based pupil perimetry method (i.e., VRgcFPP) in detecting visual field defects of patients suffering from homonymous hemianopia by assessing sensitivity and test–retest reliability.

## Methods

### Study design and subjects

This cross-sectional cohort study included human patients suffering from absolute homonymous visual field defects due to neurological impairment who completed both a gcFPP and a VRgcFPP test on two separate occasions. Information on demographic characteristics included age, sex, visual acuity, diagnosis and medication (for details, see Results). The study also included healthy controls who were tested once. The latter group were asked about any ophthalmologic problems prior to participation but did not receive ophthalmologic screening. To rule out unilateral spatial neglect, all patients were tested with the Star Cancellation Test, for which they all tested negative.

### Apparatus and stimuli

The apparatus used for the presentation of stimuli and measurement of pupillary responses have been described extensively in previous studies. For a more detailed description of apparatus and stimuli, please refer to [21, 33] for the gcFPP method and [23] for the VRgcFPP method.

**Fig. 1:**
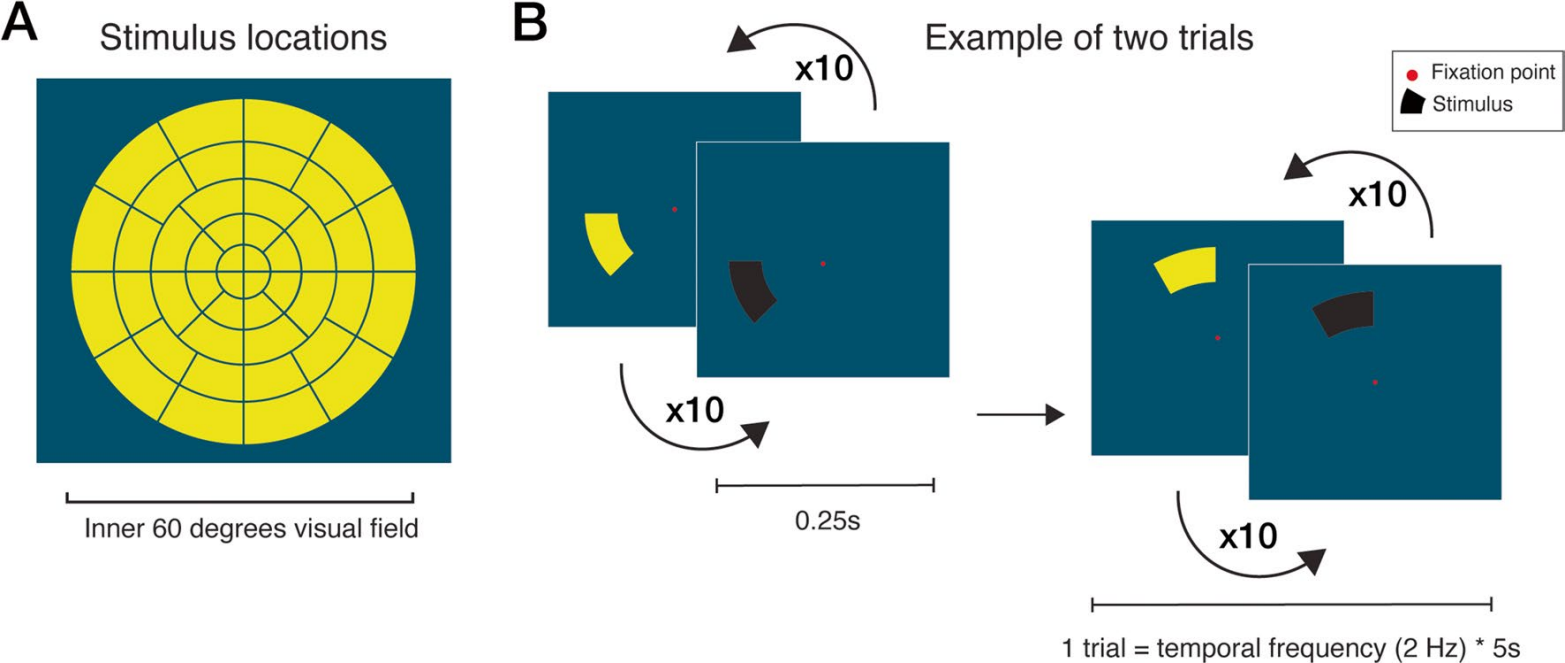
Panel (**A**) depicts all 44 stimulus locations located within the inner 60 degrees of the visual field used in both experiments. An example of two consecutive trials is shown in (**B**). A trial consisted of a single stimulus location flickering yellow-and-black at a 2 Hz rate for 5 s in a gaze-contingent manner

The stimuli consisted of black-yellow flickering wedges presented across 44 stimulus locations in randomized order within the inner 60 degrees field of vision, superimposed on a dark blue background (30% luminance for an optimal trade-off between luminance and color contrast; see [21] and positioned around a red fixation target (see Fig. 1). The gaze-contingent stimulus presentation (i.e., the eye tracking software follows the subject’s direction of gaze fixation and updates the position of the flickering stimuli real-time to reflect changes in direction of gaze) ensured stable retinotopic stimulation despite the presence of saccades [20]. A single test consisted of 220 s (44 stimulus locations * 5 s per location), excluding instruction and calibration, which consisted of a 5-point calibration grid. During the gcFPP method, only the right eye was measured while the left eye was occluded. For the VRgcFPP method, pupils were measured binocularly to estimate convergence and thus focus of depth in the VR environment. However, only data of the right eye were analyzed to allow comparison with the non-VR gcFPP version. The dual OLED screens allowed a sense of depth to prevent VR-induced simulator sickness.

### Analysis

The pupillometry analysis was identical in both pupil perimetry methods. First stimulus location onsets functioned as start events for the event-related analysis of the continuous pupil output of the integrated eye tracker. From the pupil data, blink episodes were detected and removed using an automated detection blink method by looking for crossings of a speed threshold of 4 standard deviations (SD) above the mean. The removed blink epochs were interpolated with a cubic method. Next, pupil data were baseline-corrected to enhance inter-subject comparability, to remove steady and slow-changing trends in pupil size, and to produce pupil size patterns that oscillated around zero; a necessity for proper frequency analyses. A high-pass Butterworth filter (3rd order, 1 Hz cut-off frequency) and a low-pass filter (3rd order, 10 Hz cut-off frequency) followed to remove average pupil size, slow pupil diameter changes, and high-frequency noise all together. Pupil traces per stimulus location were converted to power values in the frequency domain using a fast Fourier transform. The power at 2 Hz reflected the pupil oscillation amplitude and served as the main dependent variable per stimulus location (i.e., 44 amplitudes per participant and per perimetry method). This measurement will henceforth be referred to as the pupil response amplitude. The amplitudes were z-normalized to allow comparison across participants. Z-normalization was accomplished by first subtracting the mean and then dividing by the standard deviation of pupil amplitudes across locations per participant and perimetry method. Next, each of the 44 amplitudes per participant were subtracted with the mean and divided by the standard deviation to end up with z-scored amplitudes.

The most recent standard automated perimetry (SAP) results served as ground truth to create binary perimetry maps per patient; all stimulus locations were scored (0 = damaged, 1 = intact) to create a dichotomous outcome for analysis. The scores were based on significant Total Deviation *p*-values in the case of HFA 30–2 and the V4 stimulus pattern in the case of Goldmann perimetry (note that the Goldmann perimeter does not allow a fully automated procedure, but we stick to the abbreviation SAP for convenience) and were given while blinded to the pupil perimetry results.

To control for any potential over- or underestimation of VFDs, z-score normalized pupil response amplitudes per stimulus location and per subject were adjusted for supero-inferior and temporo-nasal visual field anisotropies (i.e. physiological increases in pupil responsiveness in the center of the VF as opposed to decreased responses in the periphery, and stronger pupil responses in the upper and temporal than lower and nasal VFs) [11, 20, 34–38]. The mean differences in the z-score normalized pupil response amplitudes between the upper-lower and temporal-nasal visual fields of healthy controls were subtracted from the z-score normalized pupil response amplitudes of participants for upper and temporal regions, respectively.

Diagnostic performance was evaluated using three statistical outcomes. The first method calculated the area under the curve (AUC) of the receiver operating characteristics (ROC) with the binary SAP maps as dichotomous independent variable that divided the 44 pupil response amplitudes (i.e., per VF location), as the dependent variable, in two distribution: an intact (visible) versus damaged (not visible) distribution. The distribution of pupil amplitudes for stimuli presented in intact versus defect locations served as “signal” and “no signal”, respectively. The ROC was produced by calculating the proportion hits versus false alarms for each pupil response amplitude threshold. The AUC below this curve produces a single score per subject. An AUC score of more than 0.5 indicates that the pupil responded stronger to stimuli presented in intact than damaged regions, where an AUC of 1.0 indicates that the response amplitudes enabled a perfect segregation between intact versus damaged regions. A score below 0.5 means that the pupil responded stronger to stimuli presented in damaged than intact locations, which may occasionally be observed if either SAP or PP produces unreliable measurements. To inspect which regions linked to weak versus strong amplitudes, normalized two-dimensional pupil sensitivity maps were created as graphical visualizations of visual field defects, with black, red, orange, and white regions indicating weakest, weak, strong, and strongest pupil responses (i.e., negative and positive z-scores), respectively.

The second method compared pupil perimetry results of patients to healthy controls. Again by using signal detection theory (i.e., calculating AUCs of ROCs), the difference between pupil response amplitudes in the intact versus damaged visual field (as determined by SAP-converted binary maps) of patients were compared against the difference in pupil response amplitude of all healthy controls in corresponding visual field locations. Note that the ROC was the result of hits and false alarm rates calculated by comparing one averaged difference in pupil amplitude of a patient to multiple averaged differences of healthy controls. An AUC score of above 0.5 means that patients showed stronger differences in pupil response amplitudes across the visual field than healthy controls, allowing for the statistical detection of visual field defects through the inspection of amplitudes.

The third method consisted of the calculation of test–retest reliabilities between the first and second measurement per patient. This was estimated using Pearson’s correlation coefficients (*r)* of the proportion increase in z-normalized pupil response amplitudes of intact visual fields as compared with amplitudes measured in damaged visual fields, per stimulus location and per patient. The proportion was calculated to mitigate any changes in the variability in pupil response amplitudes across sessions (e.g., a patient may show much weaker responses in a second session due to fatigue). The calculation consisted of first subtracting the amplitude averaged across damaged regions from intact regions, and then dividing this by the amplitude averaged across damaged regions. A proportion value above 1 indicates that z-normalized pupil response amplitudes were stronger for intact than damaged regions.

One-sample double-sided Student’s t-tests (post-hoc tests) determined statistical significance of discriminative performance (intact versus damaged or patient versus control) of pupil perimetry by comparing whether the derived AUC values differed significantly from 0.5 (baseline for no discriminative power). Paired (within-subject comparison) double-sided t-tests determined whether these AUC values differed between pupil perimetry methods. All analyses were performed using MATLAB software (version R2021b, MathWorks, Natick, MA). Raw data and analyses were made publicly available on open science framework: https://osf.io/6tcva.

## Results

### Exemplary subject

**Fig. 2:**
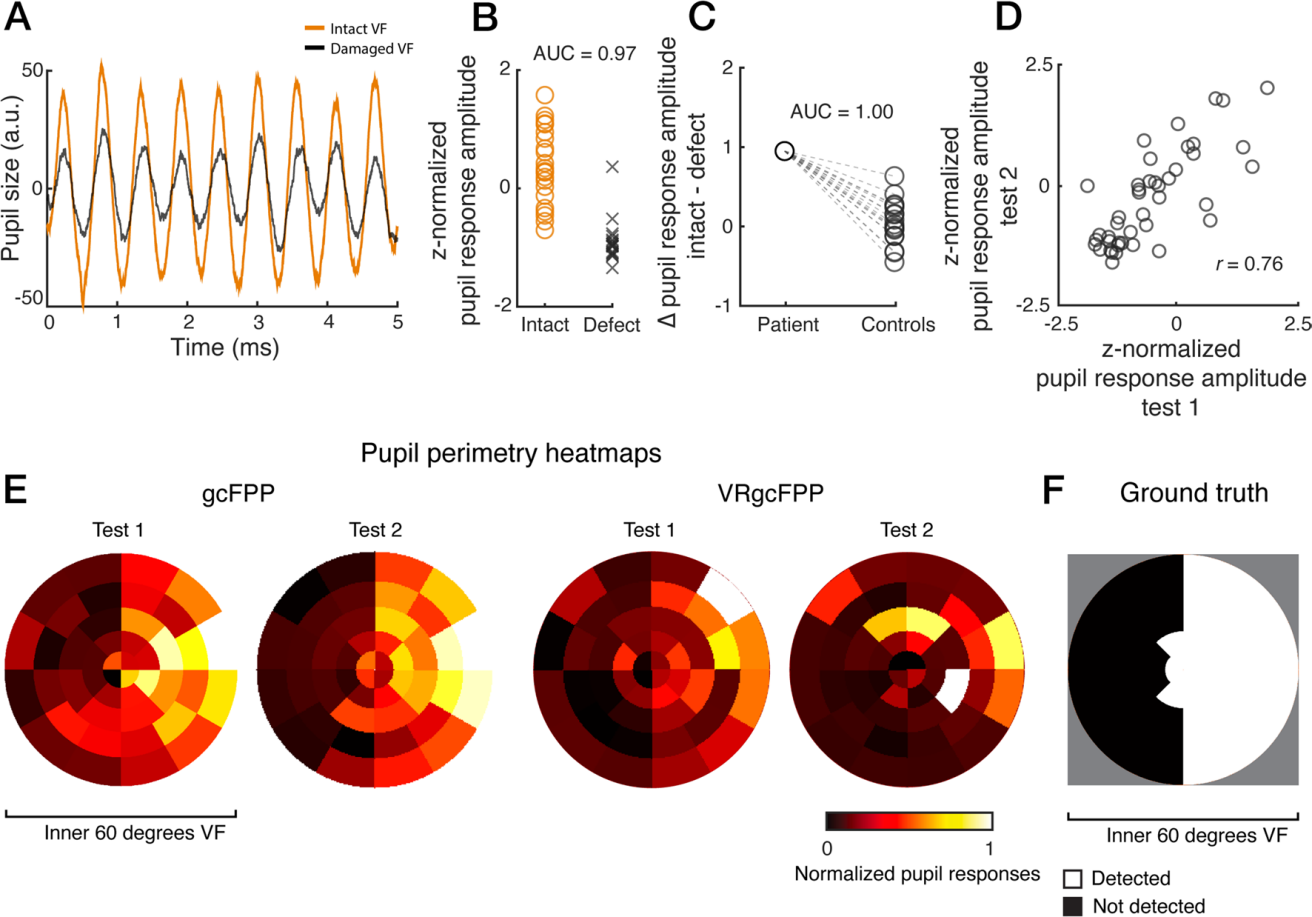
Results of exemplary subject. Panel (**A**) shows the average pupil size (in arbitrary units) in response to stimuli located in the intact (orange) and damaged (black) visual field (VF) over time. Z-normalized pupil response amplitudes per intact (orange circles) and defect (black crosses) VF location are plotted in panel (**B**). Panel (**C**) shows the average difference in pupil response amplitudes between the intact and defect visual field locations and the average difference in the corresponding visual fields per healthy control. The z-normalized pupil response amplitudes for test 1 and 2 of all 44 stimulus locations are plotted in (**D**), on which the test–retest reliability (Pearson’s correlation coefficient *r*) was based. Panel (**E**) shows two-dimensional heatmaps of normalized pupil responses of the inner 60 degrees of the VF indicating pupil sensitivities per stimulus location (weak sensitivity: red to black, strong sensitivity: yellow to white) for the gcFPP and VRgcFPP methods. Lastly, (**F**) shows the converted standard automated perimetry result which served as a binary division (black = damaged VF, white = intact VF) for (**A**), (**B**), and (**C**)

The results of an exemplary patient bundled in Fig. 2 serve to illustrate how the general diagnostic performance was evaluated. This figure shows that a pupillary oscillation in response to a flickering stimulus weakens if presented in a damaged VF location (Fig. 2A; damaged locations were determined beforehand with SAP). The normalized pupil oscillation amplitude weakened for almost every damaged as opposed to intact VF locations (Fig. 2B). In fact, damaged and intact VF locations could be differentiated based on pupil amplitudes very well, with a within-subject diagnostic accuracy (AUC; for details, see Methods) of 0.97.

Next, we looked into the discrimination of a patient from healthy subjects through the inspection of the size of the average difference between intact and damaged VFs as a between-subject diagnostic accuracy calculation. As such, the difference between pupil response amplitudes for the intact and damaged VF locations (according to SAP) of a patient was compared to the difference in pupil response amplitudes of the corresponding VF locations of all 17 healthy controls (see Fig. 2C). The difference in pupil response amplitudes of the exemplary patient was larger than the difference in all healthy controls (AUC = 1.0). This indicates that whenever a subject expresses larger than normal differences in pupil amplitudes across VF locations, this subject is likely to suffer from a VF defect.

As another analysis step, test–retest reliability was evaluated by reinviting patients and repeating the tests. The pupil response amplitudes across all 44 stimulus locations were compared between the two separate testing occasions (Fig. 2D). The correlation of pupil responses to the stimulus locations across test 1 and 2 for the exemplary subject was fairly strong (*r* = 0.76), indicating that stimuli presented in damaged VF locations reliably produced weakened pupil responses across repeated tests.

Lastly, we inspected whether a two-dimensional heatmaps of pupil response amplitudes of the VF could help to map out the defects. Such maps were plotted for tests 1 and 2 of both the gcFPP and the VRgcFPP methods for comparison with SAP results (Fig. 2E-F). The imaged patterns of SAP and pupil perimetry roughly matched, with more convincing results for gcFPP (see Supplementary Fig. S1 for the results of gcFPP, VRgcFPP and converted SAP for all patients).

### Overall diagnostic performance across patients

**Fig. 3:**
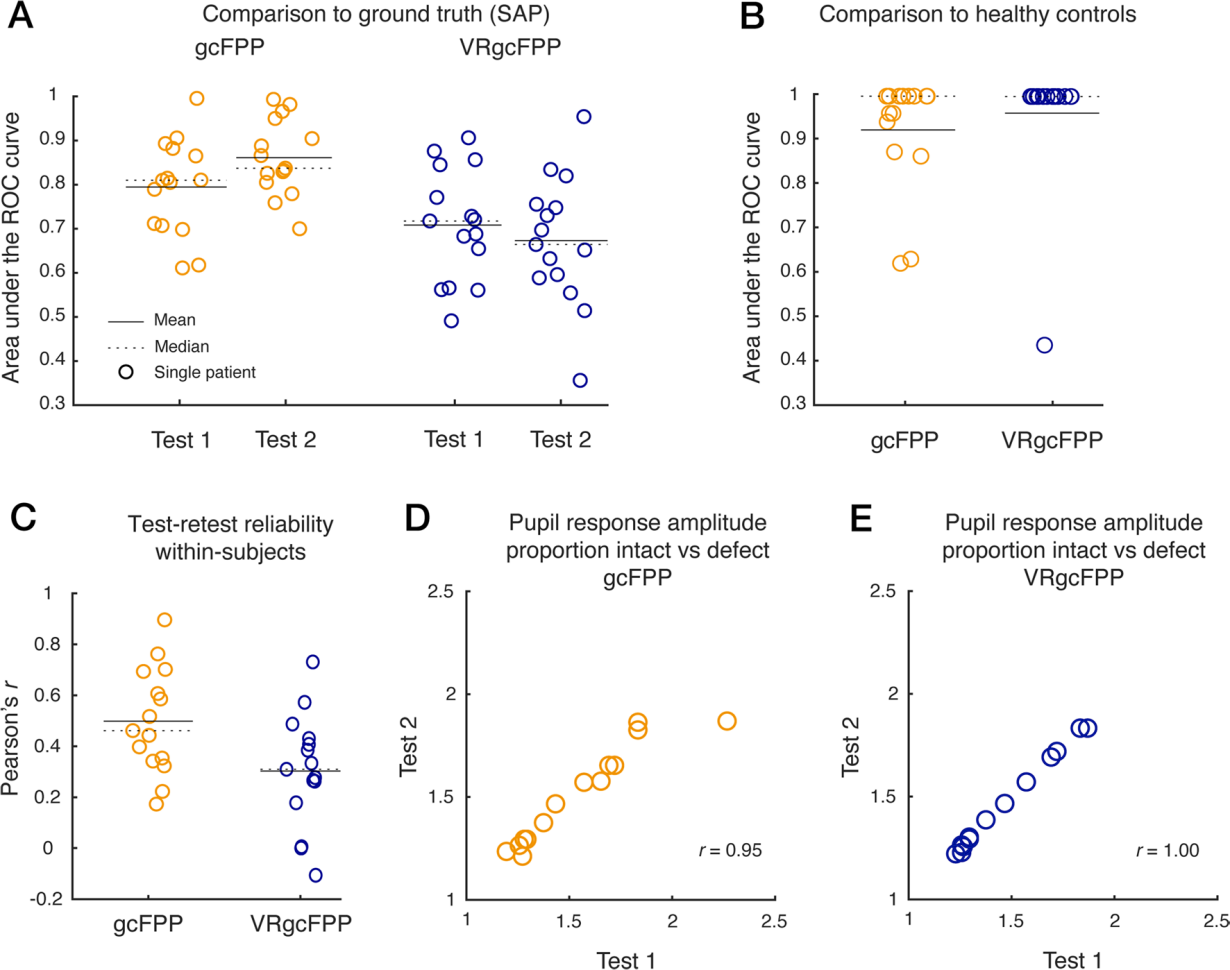
Diagnostic performance of the gcFPP (orange) and VRgcFPP (blue) methods. Panel (**A**) shows the area under curve (AUC) of the receiver operating characteristics (ROC) per patient (circles), method (color) and test session. The ROC was based on comparing the distributions of pupil amplitudes evoked by stimuli presented in intact versus damaged regions. The binary segmentation of the visual field was based on standard automated perimetry (SAP) results that were converted to binary maps (see Fig. S1). Next, we calculated the difference in pupil amplitudes between intact and damaged regions and panel (**B**) depicts the AUC of ROCs that resulted from the comparison of these differences between patients and healthy controls per method. Panel (**C**) shows test–retest reliabilities (Pearson’s correlation coefficient *r*) of pupil response amplitudes evaluated across all visual field regions per patient. Similarly, test–retest scores were examined for the proportion increase in pupil response amplitude to stimuli presented in intact as compared with damaged regions, per patient, and per gcFPP (**D**) and VRgcFPP method (**E**)

Fifteen patients (12 males, mean age (M_Age_) = 57, standard deviation (SD_Age_) = 15) and seventeen controls (6 males, M_Age_ = 53, SD_Age_ = 12) were included in this assessment. Tests 1 and 2 for the patients were on average 160 days (Mdn_Days_ = 159, SD_Days_ = 158) apart. See the Supplementary Table S1 for all patient characteristics. As in the previous result section, within-subject diagnostic accuracy was evaluated first (Fig. 3A). When averaging the results across all patients, the gcFPP method (AUC test 1: M = 0.79, SD = 0.11, compared to AUC = 0.5: *t*_14_ = 4.97, *P* < 0.001, AUC test 2: M = 0.86, SD = 0.09, *t*_14_ = 6.75, *P* < 0.001) performed better than the VRgcFPP method (AUC test 1: M = 0.71, SD = 0.13, *t*_14_ = 5.80, *P* < 0.001, AUC test 2: M = 0.67, SD = 0.15, *t*_14_ = 4.21, *P* = 0.001) in dissociating intact from defect VF locations, and this difference was statistically significant (*t*_14_ = 2.34, *P* = 0.04). More importantly, Fig. 3B shows that gcFPP and VRgcFPP performed comparably in discriminating patients from healthy controls (AUC gcFPP: M = 0.92, SD = 0.13, compared to AUC 0.5: *t*_14_ = 12.29, *P* < 0.001; AUC VRgcFPP: M = 0.96, SD = 0.15, *t*_14_ = 11.93, *P* < 0.001).

Each method’s reliability was assessed by comparing pupil response amplitudes across tests 1 and 2 of gcFPP and VRgcFPP, respectively (see Fig. 3C). A moderate-to-low within-subject correlation (gcFPP M = 0.50, SD = 0.21; VRgcFPP M = 0.30, SD = 0.22) indicates that the overall pupil’s sensitivity to stimuli (i.e., the pupil’s baseline state to respond less or more vigorously) varies across testing sessions despite comparable testing conditions. To mitigate such baseline effects that interfere with the reliability assessments, we calculated the proportion pupil response amplitudes (for details, see Methods). However, when considering the proportion of pupil response amplitudes in intact versus damaged visual fields a very high test–retest reliability is found (gcFPP *r* = 0.95, *P* < 0.001, VRgcFPP *r* = 1.00, *P* < 0.001; see Fig. 3D and 3E). Overall, these results can be summarized as follows: (1) the comparison of pupil response amplitudes between intact versus damaged regions per patient indicate that gcFPP allows for cleaner imaging of intact versus damaged visual field regions than VRgcFPP, (2) the comparisons of average differences in intact versus damaged amplitudes between patients and controls demonstrate high diagnostic performance of both gcFPP and VRgcFPP, and (3) the test-rest reliabilities confirm that both gcFPP and VRgcFPP reliably and consistently measures defects in neurologically impaired patients.

## Discussion

We assessed the diagnostic performance of gaze-contingent flicker pupil perimetry (gcFPP) in an original campimetry-like setting and a virtual reality setting (VRgcFPP) in a cohort of adult patients suffering from homonymous visual field defects (VFD) due to neurological impairment. Compared to other pupil perimetry methods [12–19], this study reports the highest diagnostic accuracy so far. Other methods, such as Matrix frequency doubling technology perimetry or multifocal visual evoked potentials, have been introduced in an effort to accurately and reliably evaluate VFs in neurologically impaired patients [39–46], but all exhibited caveats which retained it from adoption in common practice or simply did not reach the diagnostic accuracy of SAP. The current methods’ diagnostic performances and ease of use, warrants it as a valuable addition to VF assessment when SAP is unproductive (e.g. the patient is too young, neurologically impaired or suspected of malingering).

Moderate repeatability of pupil response amplitudes between stimulus locations supports the notion that the pupil expresses baseline variability across time evoked by many factors that modulate cognitive and (para-) sympathetic nervous systems [9, 11, 47–49]. However, a stronger test–retest reliability (or low test–retest variability) is found for the relative increase in response amplitudes for intact versus damaged (i.e., proportion) visual fields across all patients. Although direct comparisons are required in future studies, this may indicate that the objective pupil perimetry method is more reliable in diagnosing large within-subject deviations in sensitivity across the visual field than SAP, which have been found to have significant intertest threshold variability [3, 4]. Importantly, half of the patients were tested after a year and the other half after a few days. This dissociation does not impact test–retest reliability. Although only a small cohort was tested, it contributes to the notion that the pupil is a robust measure of visual field sensitivity. Previous literature shows that SAP methods suffer from high test–retest variability [3, 4]. Reported Pearson’s correlation coefficients of test–retest reliability of SAP range between 0.20 and 0.87 for homonymous hemianopia and glaucoma, respectively [50, 51]. In healthy children aged 8 years and upwards, test–retest reliability is reported to be as low as 0.35 [52]. The poor reproducibility might be caused by retinotopic displacement of small stimuli due to the fixational jitter or microsaccades, and learning and fatigue effects [5–7]. Furthermore, test–retest variability of SAP has been shown to be larger if visual field sensitivity is reduced, which is the case in patients with homonymous hemianopia. The variability of a single threshold estimate is even nearly equivalent to the dynamic range of the instrument for damaged locations [53]. In one study the test–retest variability of SAP was 127% higher in locations with 20-dB loss compared to locations with 0-dB loss [54]. For gcFPP, test–retest variability and noise in the visual field heatmaps might also partly be due to small variations in eye tracker calibration. The placement of a stimulus on the edge of a scotoma might also influence noise of visual field heatmaps.

Pupil perimetry shows similar potential in diseases such as age-related macular degeneration [55, 56], diabetic retinopathy [57] and retinal and optic nerve diseases [58], but shows varying performance in glaucoma [20, 59], even when using a head mounted perimeter [26, 60]. However, it may be argued that pupil perimetry could be epileptogenic in the more at-risk neurologically impaired patient due to the flickering stimuli. Luckily, a recent paper shows that, despite the flickering stimuli used in pupil perimetry, it is even possible to assess patients with epilepsy without inducing seizures although this method discriminated patients from controls with considerably lower performance [61].

The importance of assessing the VF in young and neurologically impaired individuals and the poor reliability of SAP in this population [1, 62, 63] stress the need for an objective alternative. The (VR)gcFPP methods show promise by capably distinguishing patients from healthy controls. Interestingly, the VR method was not as accurate as its gcFPP counterpart. The latter used a more sophisticated eye-tracker, possibly explaining the better signal-to-noise ratio as evident in the visual field heatmaps (see Supplementary Fig. S1). In other words, VRgcFPP is a capable screening method to investigate whether an individual suspected of a VFD deviates from healthy controls, but it is difficult to pinpoint the exact location of the defect. The non-VR version will produce more promising results for the latter. This does not mean that VRgcFPP cannot be of use in practice. In fact, by changing the stimulus locations to, for example, only four quadrants, a very rough though fast visual field estimate can be realized for screening purposes. Additionally, the introduction of a small gap between stimulus locations might facilitate a more accurate comparison between results from pupil perimetry and current SAP methods by providing a similar step in sensitivity.

This is not the first time that head-mounted VR technology is harnessed in an attempt to improve feasibility of VF assessment [24–26, 28, 29, 32, 64]. Despite its many advantages, this relatively young technique remains to be perfected. Future development may lead to the implementation of eye-trackers with better precision to reduce noise and subsequently increase accuracy.

This study is limited by the use of retrospectively gathered (most recent) perimetry results from varying perimeters (i.e. Goldmann and HFA). Prospective SAP testing with one perimetry method and fixed settings consistently across patients would allow for better comparisons. Also, while this study shows pupil responses to stimuli shown in intact VF regions are stronger than those shown in damaged regions, the current visual field heatmaps do not strongly resemble SAP test results for all patients. Currently, gcFPP is likely more suited to initial detection of visual field defects as a screening tool rather than clinical follow-up of disease progression. Another limitation consists of the binocular testing during VRgcFPP as it might cause issues in patients unable to accommodate. It is currently not possible to stimulate only one eye in the virtual reality environment, leading to the measurement of combined direct and consensual responses. A future version enabling monocular testing might increase diagnostic accuracy. Finally, more thorough assessment of cognitive abilities on top of the Star Cancellation Test, such as with the MoCA or TOSSA tools [65, 66], might be interesting to include in future studies.

To summarize, gcFPP and VRgcFPP are fast and reliable methods with robust measurements. The VRgcFPP method in particular is cheap and offers a mobile solution, at the sacrifice of some accuracy, for those patients unable to restrict their head movements for prolonged time periods. We recommend the use of these two pupil perimetry methods as complementary tools to the standard visual field work-up of neurologically impaired individuals.

## Supplementary Information

Below is the link to the electronic supplementary material.

Supplementary file1 (DOCX 546 KB)
