# Supplementary material for: Diagnostic performance of pupil perimetry in detecting hemianopia under standard and virtual reality viewing conditions
Source: Graefes Arch Clin Exp Ophthalmol. 2024 Sep 18;263(2):537–46. doi: 10.1007/s00417-024-06641-4 (PMC11868179; doi:10.1007/s00417-024-06641-4)
Supplement: Supplementary file 1 — Supplementary file1 (DOCX 546 KB) [file 417_2024_6641_MOESM1_ESM.docx]

# Supplementary materials

| Patient | Age | Gender | VA with correction (logMAR) | Days between tests | Visual field defect | Diagnosis | Medication |
| --- | --- | --- | --- | --- | --- | --- | --- |
| s1 | 65 | Male | -0.1 | 327 | RHH | Stroke left occipital cortex | Clopidogrel, ezetimibe, nifedipine, pantoprazole, simvastatine, tamsulosine |
| s2 | 56 | Male | 0 | 334 | ILQ | Stroke right occipital cortex | Acenocoumarol, acetylsalicylic acid, amlodipine, citalopram, metoprolol, perindopril, rosuvastatin |
| S3 | 28 | Male | -0.1 | 336 | RHH | Stroke left occipital cortex after tumor resection left frontal cortex | Levetiracetam |
| S4 | 71 | Male | 0 | 329 | ILQ | Stroke right occipital cortex | Simvastatin, apixaban, levetiracetam, hydrochlorothiazide, digoxin, losartan, tramadol |
| S5 | 57 | Male | -0.1 | 369 | LHH | Stroke right occipital cortex | Metoprolol, pantoprazole, rosuvastatin, lisinopril, desloratadine |
| S6 | 68 | Male | 0 | 309 | RHH | Stroke left occipital cortex | Acetylsalicylic acid, simvastatin |
| S7 | 74 | Male | 0 | 208 | LHH | Stroke right occipital cortex | Perindopril, rosuvastatin, clopidogrel, hydrochlorothiazide, amitriptyline, pantoprazole |
| S8 | 79 | Male | 0 | 159 | LHH | Stroke right occipital cortex | Atorvastatin, acetylsalicylic acid |
| S9 | 49 | Female | 0 | 7 | LHH | Stroke right occipital cortex | Amlodipine, candesartan, spironolactone |
| s10 | 46 | Male | 0.05 | 7 | RHH | Stroke left occipital cortex | Acenocoumarol, formoterol, levocetirizine |
| s11 | 49 | Female | -0.1 | 8 | SRQ | Stroke left occipital cortex | Clopidogrel, simvastatin |
| S12 | 63 | Male | 0 | 2 | LHH | Stroke right occipital cortex | Atorvastatin, clopidogrel, amlodipine, lisinopril |
| S13 | 67 | Male | 0 | 2 | ILQ | Stroke right occipital cortex | Clopidogrel, pantoprazole, pravastatin |
| S14 | 55 | Female | 0 | 7 | SRQ | Stroke left occipital cortex | Valsartan |
| S15 | 28 | Male | -0.2 | 7 | LHH | Stroke right occipital cortex after resection arteriovenous malformation | Mebeverine, fexofenadine |

**Table S1** Patient demographics. VA = visual acuity (in logMAR); LHH = (partial) left homonymous hemianopia; RHH = (partial) right homonymous hemianopia; ILQ = inferior left homonymous quadrantanopia; SRQ = superior right homonymous quadrantanopia.


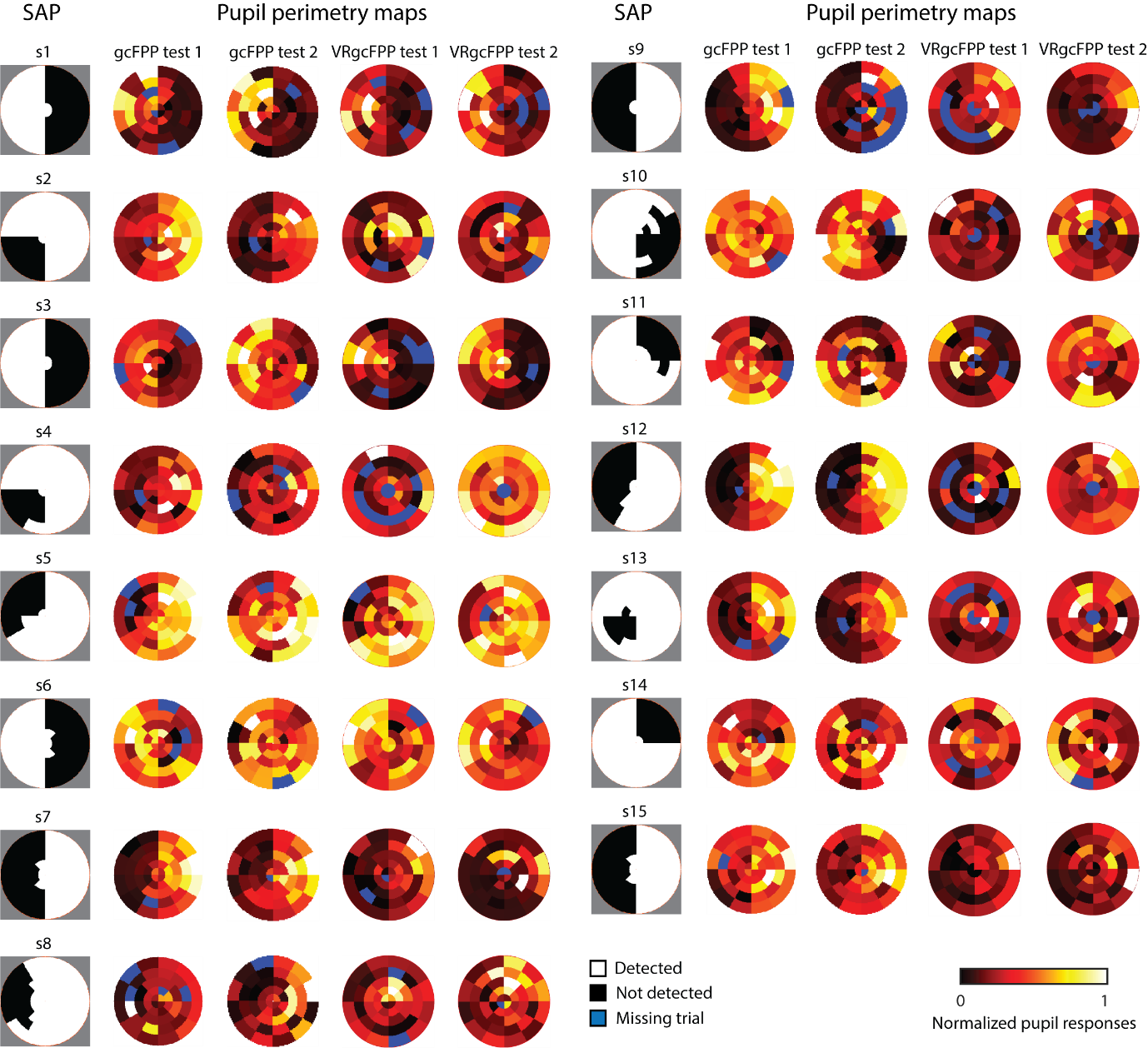


**Fig. S1 Visual field maps of all patients and tests.** The first column shows the ground truth based on standard automated perimetry (SAP; white = detected, black = undetected stimuli during Goldmann kinetic perimetry or Humphrey Field Analyzer testing of the inner 60 degrees of the visual field) per participant (s1-s15). The next four columns show the two-dimensional heatmaps of normalized pupil responses for tests 1 and 2 of the gaze-contingent flicker pupil perimetry (gcFPP) and the virtual reality (VR) gcFPP methods.
